# The Role of the Anterior Temporal Lobe in Reading: An HD-tDCS Study

**DOI:** 10.1162/NOL.a.266

**Published:** 2026-07-01

**Authors:** Sophie Arheix-Parras, Cheng Xiao, Sidney Crouse, Nicholas Riccardi, Karim Johari, Rutvik H. Desai

**Affiliations:** Department of Psychology, University of South Carolina, Columbia, SC, USA; Linguistics Program, University of South Carolina, Columbia, SC, USA; Human Neurophysiology and Neuromodulation Lab, Communication Sciences and Disorders, Louisiana State University, Baton Rouge, LA, USA; Institute for Mind and Brain, University of South Carolina, Columbia, SC, USA

**Keywords:** ATL, lexical, reading, semantic, tDCS

## Abstract

The anterior temporal lobe (ATL) is suggested as a semantic hub that may support reading inconsistent words via semantic access. Surface alexia is characterized by difficulty in reading these inconsistent words, and often co-occurs with ATL atrophy and semantic impairments. However, the role of ATL in word reading is unclear, as evidenced by cases of alexia without semantic impairments and semantic impairments without surface alexia. To test its role in reading, we stimulated the ATL using transcranial Direct Current Stimulation in neurotypical participants performing a Word Naming task including consistent and inconsistent words, and a Picture Plausibility (PP) task involving nonverbal semantic judgments. We also stimulated the left tempo-occipital cortex (TOC) during the PP task. Low-frequency inconsistent words were read more slowly than high-frequency inconsistent words in the sham condition. This interaction disappeared with ATL stimulation. We found an interaction between consistency and stimulation in the low-frequency subset, but not in the high-frequency subset. In the PP task, ATL stimulation had no effect, whereas TOC stimulation significantly influenced reaction time. These findings support the left ATL’s role in reading inconsistent words, aligning with surface alexia with ATL atrophy. The results also suggest that the left lateral ATL may be involved in lexical rather than purely semantic processes, consistent with lesion studies demonstrating a dissociation between semantic impairments and surface alexia, and possibly also with graded modality-specific specialization of bilateral ATLs. These findings reconcile conflicting findings and elucidate the role of left lateral ATL in reading.

## INTRODUCTION

The capacity to map spelling to sounds is an essential human cognitive skill. Behavioral work has shown that consistency, reflecting spelling-to-sound mapping, plays an important role in reading (Chee et al., 2020; Seidenberg et al., 1984; Strain et al., 1995; Taylor et al., 2011). Models of word reading (e.g., Coltheart et al., 2001; Plaut et al., 1996) agree that pseudowords rely on sub-lexical processes, while inconsistent or exception words require contribution from different pathways involving either whole-word or semantic processing. The dorsal phonological/sub-lexical pathway activates left areas including the insula, the inferior frontal gyrus, the inferior parietal cortex and occipitotemporal regions. A ventral lexicosemantic pathway is thought to involve the left middle temporal gyrus, the inferior-temporal sulcus, and the bilateral angular gyrus (Graves et al., 2010; Jobard et al., 2003; Taylor et al., 2013). Several studies implicate the anterior temporal lobe (ATL) in lexicosemantic processes of word reading, particularly for low-frequency inconsistent words (Hoffman et al., 2015; Provost et al., 2016; Wilson et al., 2012). Indeed, an fMRI study highlighted the crucial role of the left ATL in reading aloud inconsistent words (Hoffman et al., 2015). Two repetitive Transcranial Magnetic Stimulation (rTMS) studies targeting the left ATL reported effects on inconsistent word reading (Ueno et al., 2018; Woollams et al., 2017). More specifically, Woollams et al. (2017) showed that processing of inconsistent words was strongly impacted in participants who rely more on semantic information when reading.

Alexia is an acquired reading impairment following brain lesions or neurodegenerative pathologies (e.g., stroke: Bahrami Balani & Bickerton, 2025; Primary Progressive Aphasia, PPA: Westbury & Bub, 1997). Surface alexia is a type of alexia characterized by difficulty in reading words with inconsistent spelling that do not follow standard grapheme-to-phoneme conversion rules (e.g., *pint*), inducing more regularization errors (e.g., the inconsistent word *crypt* read as /krɪpt/) (Ripamonti et al., 2014). The majority of people with the semantic variant of PPA (svPPA) present surface alexia (Brambati et al., 2009; Hodges et al., 1992; Jefferies et al., 2004; Patterson & Hodges, 1992; Patterson et al., 2006; Woollams et al., 2007) with atrophy usually starting in the left ATL (Teichmann et al., 2019). Indeed, surface alexia was previously a diagnostic feature (Warrington & Shallice, 1979). Two studies have suggested that compared to neurotypicals, svPPA participants with left ATL atrophy and surface alexia relied more on sub-lexical pathway regions when reading inconsistent words (Borghesani et al., 2020; Wilson et al., 2009).

These findings mesh well with the suggestion that inconsistent words are read by accessing semantics (Harm & Seidenberg, 2004; Plaut et al., 1996; Woollams et al., 2010; Woollams et al., 2007). Since ATL has been suggested as a putative semantic hub (Lambon Ralph et al., 2017; Patterson et al., 2007), surface alexia severity may be linked to semantic impairments severity in svPPA (Graham et al., 2000; Jefferies et al., 2004; Patterson & Hodges, 1992; Woollams et al., 2007).

However, some studies challenge this association between surface alexia, ATL, and semantics. Some individuals with post-stroke alexia show preserved semantic processes (Binder et al., 2016; Gvion & Friedmann, 2016), while some with PPA and semantic impairments do not show surface alexia (Blazely et al., 2005; Cipolotti & Warrington, 1995). It is worth noting that the rate of these exceptional cases is low, that the consistency effect has been observed in post-stroke participants but is less pronounced than in svPPA, and that all individuals with svPPA may develop surface alexia as the disease progresses (Binder et al., 2016; Woollams et al., 2007). Playfoot et al. (2018) found that semantic performance did not predict inconsistent word reading in svPPA and suggested that inconsistent word reading performance may depend also on damage to posterior temporal regions. Another explanation for the dissociation between semantic deficit and surface alexia in some individuals is that the degree of semantic reliance required to read inconsistent words may vary depending on interindividual premorbid differences (Hoffman et al., 2015; Woollams et al., 2007). Furthermore, the status of ATL as a semantic hub remains debated (Desai & Riccardi, 2021; Fernandino et al., 2016; Persichetti et al., 2021; Simmons & Martin, 2009).

Several non-invasive brain stimulation studies, using transcranial Direct Current Stimulation (tDCS) or rTMS, have contributed to the debate on the role of the ATL in lexicosemantic processes. Targeting the left ATL with low-frequency rTMS, Pobric et al. (2007) showed impaired performance in Naming and Synonym Judgment tasks, whereas they reported no effect on non-linguistic cognitive tasks (number naming and number quantity judgment). Jung and Lambon Ralph (2021) demonstrated that high- vs. low-frequency rTMS targeting the left ATL could, respectively, enhance or impair the reaction times in a Category Judgment Task on written words. Using tDCS to stimulate both ATLs, Binney et al. (2018) highlighted a facilitatory effect on semantic fluency, compared to two other stimulation sites (i.e., inferior parietal lobe and dorsal frontal cortex). Another rTMS study comparing left and right ATL stimulation revealed that only left stimulation increased the latencies in a Picture Naming task (Woollams et al., 2017), suggesting specialization of the left hemisphere for language production processes. Indeed, a meta-analysis proposed that both ATLs function as hubs of the semantic system, distinguished by their ability to process verbal vs. visual stimulus modalities (Rice, Hoffman, et al., 2015).

tDCS is based on the application of a small electrical current through electrodes placed on the scalp with the aim to change neuron depolarization susceptibility (Quartarone et al., 2006). Based on motor excitability, anodal tDCS is believed to induce a facilitatory effect on the targeted cortex, while cathodal stimulation will induce an inhibitory effect (Quartarone et al., 2006). However, some studies working on cognitive processes highlighted discrepancies in behavioral results and neural effect of high-definition (HD) tDCS, reporting for instance inhibitory effects with anodal stimulation (e.g., Binney et al., 2018; Kirimoto et al., 2011; Tabari et al., 2024). One hypothesis for this differential effect is the orientation of the neuron population relative to the current flow that can lead to inhibition or facilitation using the same anodal or cathodal stimulation (Li et al., 2015; Rahman et al., 2013). Another hypothesis for the opposite effect relies on the task difficulty, with high demanding tasks inducing stronger effect of the stimulation (Berryhill et al., 2014; Framorando et al., 2021; Wang et al., 2021). Finally, some authors have suggested that for HD-tDCS, anodal/cathodal terminology should not be used as it does not necessarily correspond to excitatory/inhibitory stimulation (Garnett et al., 2015).

The present study used HD-tDCS to examine the role of left ATL in word reading processes and semantics in three experiments. We hypothesized that ATL stimulation will differentially affect low-frequency inconsistent words. Focusing on inconsistent words allowed us to examine ATL’s role in processing these words and to highlight the potential involvement of semantic processes. A nonverbal semantic task was used to test ATL’s potential role in semantic processing not involving a lexical component. If tDCS targeting the ATL modulates both inconsistent word reading and nonverbal semantic judgments, it would support a link between inconsistent word reading, ATL, and semantics. If only inconsistent word reading is modulated, it would suggest that ATL’s role in reading is not related to semantic access. As a control experiment, we stimulated the tempo-occipital cortex (TOC). TOC is thought to be a “lexical interface,” connecting phonology to semantics in some models (Coltheart et al., 2010; Hickok & Poeppel, 2007). At the same time, it is also strongly associated with visual-semantic processing and expected to affect nonverbal semantic processing (Bracci et al., 2015; Fairhall & Caramazza, 2013; Kable et al., 2002; Kellenbach et al., 2003; van Dam et al., 2019). For example, Fairhall and Caramazza (2013) showed that representations in TOC contain feature-based similarity structure using Representational Similarity Analysis on object pictures from five semantic categories. van Dam et al. (2019) showed that manner and instrument verb pictures, in a nonverbal semantic plausibility task, can be classified using signal from the TOC. Hence, we used TOC as a candidate region where stimulation can be expected to affect nonverbal visual-semantic judgments.

## METHODS

### Participants

We included 83 neurotypical participants (67.2% female, mean age = 20.8; *SD* = 1.7; age range = 18–25). All participants were native English speakers, right-handed according to the Edinburgh Handedness Inventory (Oldfield, 1971) and had normal or corrected-to-normal vision and hearing. Written consent was obtained from all participants who received monetary compensation or extra course credit for their participation. The study was approved by the Institutional Review Board of University of South Carolina.

We report results from three experiments using two different tasks. For Experiment 1, 34 participants received stimulation targeting the left ATL and performed a Word Naming task (described below). In Experiment 2, 39 participants received stimulation in the left ATL and performed a Picture Plausibility task (with 18 participants being common to both Experiments 1 and 2). In Experiment 3, 28 participants received stimulation targeting the left TOC and performed the Picture Plausibility task. There was no overlap in subjects between Experiment 3 and either Experiment 1 or Experiment 2.

All participants underwent two HD-tDCS sessions, corresponding to real and sham stimulation, with the order of stimulation counterbalanced across participants. Both sessions were separated by approximatively 3 days (mean = 3.10; *SD* = 4.71; range 1–27 days) to avoid potential carryover stimulation effects. To further control for an order effect, we analyzed the interaction effect between Stimulation Order (Real/Sham or Sham/Real) and Stimulation Type (Real or Sham) in the subset of participants who had only a 1-day gap between stimulations (*n* = 19).

### Behavioral Tasks and Stimuli

#### Word Naming task

Experiment 1 used the Word Naming task. The stimuli were presented on a screen for 4,500 ms using E-Prime 2.0 software (Psychology Software Tools). Participants were instructed to read them aloud as quickly and accurately as possible. Participants completed 10 practice trials before the task began.

The task included 220 stimuli: 80 consistent words (e.g., *cave*), 80 inconsistent words (e.g., *gym*), and 60 pronounceable pseudowords (e.g., *afoub*). All consistent and inconsistent words were selected based on the database of Chee et al. (2020). All pseudowords were selected from the English Lexicon Project (Balota et al., 2007). To avoid repetition and familiarity effect across sessions, the stimuli were divided into two halves, with each half presented in one session. The two halves were counterbalanced across participants.

Several variables may have impacted RT apart from stimulation and brain target for both words and pseudowords. For the subset of words, these variables include frequency (Brysbaert & New, 2009), concreteness (Brysbaert et al., 2014), and valence (Warriner et al., 2013). For both words and pseudowords, we considered the number of letters, the average frequency of the unconstrained bigrams (Bigram_avg_U_log) (Medler & Binder, 2005), the OLD20 (Orthographic Levenshtein Distance 20), and feedforward and feedback consistency (Chee et al., 2020; Wiley et al., 2024). An unconstrained bigram refers to any two-letter combination within a word, regardless of position or word length (Medler & Binder, 2005). The OLD20 is defined as the average distance of a word to its 20 closest orthographic neighbors (Yarkoni et al., 2008).

Consistent and inconsistent words were matched in frequency, concreteness, valence, number of letters, unconstrained bigrams, but not in OLD20, as consistent words typically have more orthographic neighbors (*t* = −4.16, *p* < 0.001), nor in feedback (*t* = 4.06, *p* < 0.001) or feedforward consistency (*t* = 8.13, *p* < 0.001). The unconstrained bigrams were matched between both type of words and pseudowords, but the number of letters (*t* = −9.72, *p* < 0.001) and the OLD20 (*t* = −8.25, *p* < 0.001) differed (Table 1). Our goal was not to directly compare words to pseudowords, and while we report results for pseudowords as a separate class of stimuli here, they were included to examine independent questions not discussed here. In our analysis, we divided the word data set (comprising consistent and inconsistent words) into two subsets based on a median split: low-frequency and high-frequency words. These two subsets were matched in feedback (*t* = −1.79, *p* = 0.07) and feedforward consistency (*t* = −0.33, *p* = 0.74).

**Table 1.** Mean values and standard deviations of variables in consistent, inconsistent and pseudowords.

	**Consistent words**	**Inconsistent words**	**Pseudowords**
Number of letters	4.35 (0.64)	4.41 (0.81)	6.33 (1.41)
Bigram_avg_U_log	4.27 (0.30)	4.19 (0.37)	4.22 (0.27)
OLD20*	1.40 (0.29)	1.61 (0.34)	2.52 (0.89)
Frequency	2.73 (0.85)	2.86 (0.95)	–
Concreteness	3.76 (0.82)	3.76 (1.09)	–
Valence	5.37 (1.16)	5.48 (1.17)	–
Feedforward consistency*	0.76 (0.11)	0.60 (0.14)	0.72 (0.13)
Feedback consistency*	0.66 (0.18)	0.55 (0.18)	0.69 (0.14)

* Indicates a significant difference between consistent and inconsistent words (*p* < 0.001).

#### Picture Plausibility task

**Figure 1.**
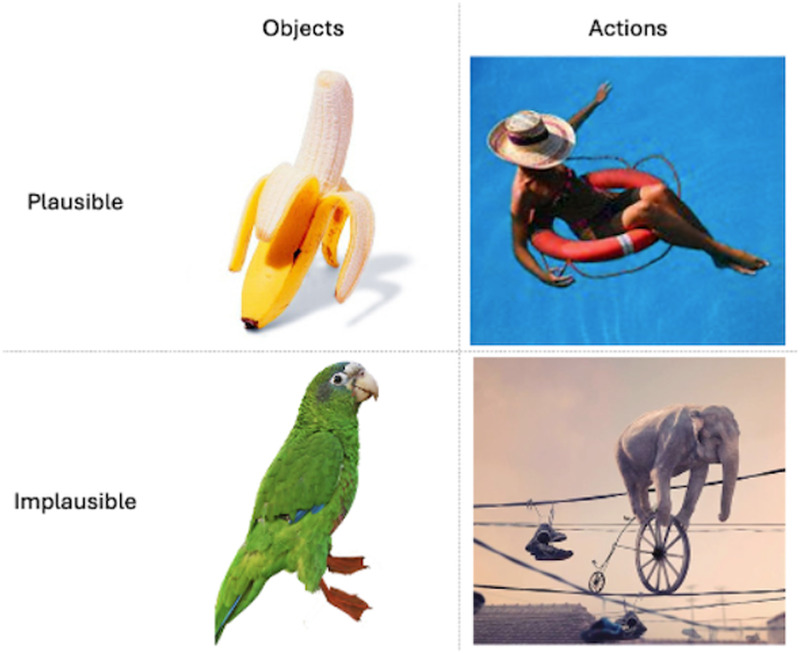
Example of a plausible object (top left), an implausible object (bottom left), a plausible event (top right), and an implausible event (bottom right).

Experiments 2 and 3 used the same task, but stimulated ATL and TOC, respectively. Pictures were presented on a screen to the participants for 4,000 ms using E-prime 2.0 software (Psychology Software Tools). Participants completed eight practice trials to ensure they understood the task. Some pictures represented plausible objects (e.g., *a banana*) or events (e.g., *floating*), while others depicted implausible objects (e.g., *a parrot with duck feet*) or events (e.g., *an elephant riding a bicycle on a power line*). Participants were instructed to press the “j” key if they thought the object or event in the picture was plausible and the “k” key if they thought it was implausible (Figure 1).

The stimulus set included 50 plausible pictures and 50 implausible pictures. To avoid repetition and familiarity across sessions, the pictures were divided into two versions, resulting in 50 stimuli per session, with half plausible and half implausible. Half of the stimuli represented objects, and the other half represented events.

### HD-tDCS Configuration

**Figure 2.**
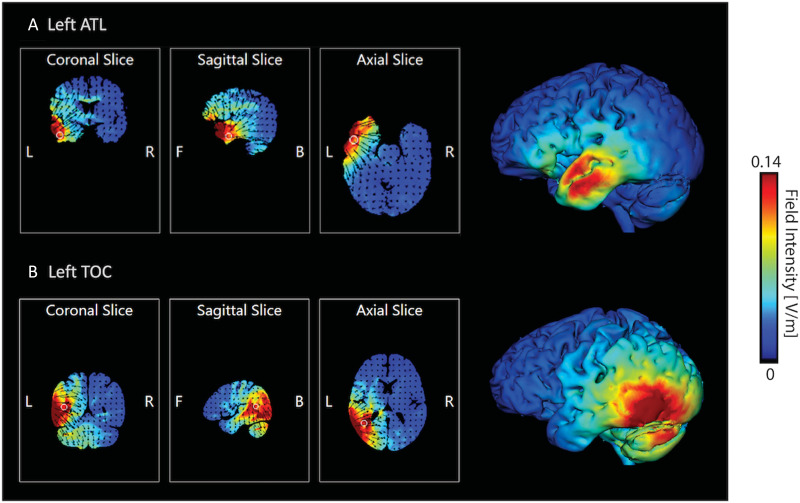
The offline model of current-induced changes in the field intensity of the anodal (A) left ATL (Experiments 1 and 2) and (B) left TOC (Experiment 3) stimulations.

In all three experiments (Word Naming task targeting ATL, Picture Plausibility task targeting ATL or Picture Plausibility task targeting TOC), participants received anodal and sham stimulations for 20 min, applied with an M × N HD-tDCS Stimulator (Soterix Medical NY, USA). Both anodal and cathodal HD-tDCS can be used to modulate brain activity. Several studies have indicated robust effects of anodal tDCS/HD-tDCS on the ATL (e.g., Papagno et al., 2021; Ross et al., 2011). In a direct comparison, anodal HD-tDCS led to more robust changes in EEG activity compared to cathodal stimulation (Tabari et al., 2024), and hence we opted for anodal stimulation here. The electrodes configurations and their corresponding current intensities are shown in Table 2 for the left ATL (Experiments 1 and 2) and left TOC (Experiment 3). HD-Explore and HD-Target software (Soterix Medical NY, USA) were used to compute electrode configurations (locations and intensities) based on the current simulation. Multiple studies comparing tDCS intensities have reported that stimulation at 1 mA led to stronger behavioral effects compared to higher or lower intensities (i.e., 0.7 mA, 1.5 mA, and 2 mA) (Ehrhardt et al., 2021; Khalil et al., 2023; Lerner et al., 2021). Figure 2 shows the electrode configurations and the modeled pattern of field intensity for anodal HD-tDCS for both stimulation targets.

**Table 2.** Electrode configurations in left ATL and left TOC.

**Left ATL**		**Left TOC**	
**Location**	**Current**	**Location**	**Current**
FT9	+1.00 mA	P7	1.00 mA
F9	−0.60 mA	PO9	−0.40 mA
FP1	−0.10 mA	O9	−0.30 mA
C1	−0.10 mA	PO7	−0.10 mA
PO9	−0.20 mA	P9	−0.20 mA

To administer HD-tDCS, a standard 10–20 EEG cap (Easy-Cap, Germany) was placed on the subject’s head, with the Cz position midway between inion and nasion, and between the two mastoids. Anodal stimulation was used as real stimulation, and the control stimulation was a passive sham involving a 60-second ramp-up at the beginning of the session and a 60-second ramp-down at the end. This sham procedure helped mimic the scalp sensations associated with real stimulation at the start, while ensuring that no current was delivered throughout the session (Davis et al., 2013; Garnett & den Ouden, 2015; Richardson et al., 2014). The practice task began after the 20 min of stimulation. To ensure that actual and sham stimulation induced similar scalp sensations, participants were asked to rate their pain and unpleasantness, 30 s after, 10 min after and 30 s before the end of each stimulation session. Evaluating the mean sensation across the three time points, they reported comparable scores of pain (ATL: F = 1.733, *df* = 1, *p* = 0.189; TOC: F = 0.699, *df* = 1, *p* = 0.410) and unpleasantness (ATL: F = 0.281, *df* = 1, *p* = 0.597; TOC: F = 0.367, *p* = 0.549) across real and sham stimulation. We also did not find any interaction effect of time and Stimulation (i.e., real or sham) for pain (ATL: F = 1.656, *df* = 2, *p* = 0.193; TOC: F = 0.875, *df* = 2, *p* = 0.427) and unpleasantness (ATL: F = 2.819, *df* = 2, *p* = 0.061; TOC: F = 1.148, *df* = 2, *p* = 0.331).

### Analysis

In the Word Naming task, trials with RT < 400 ms or > 1,500 ms for words and > 2,500 ms for pseudowords were removed. In the Picture Plausibility task, RT < 400 ms and > 2,000 ms were cleaned. In a second pass, for both tasks, outliers with RT more than three standard deviations from the participant’s mean RT were excluded. This resulted in the exclusion of 11.9% of the trials for the Word Naming task and 8.0% for the Picture Plausibility task.

For analysis, each verbal response in the Word Naming task was recorded and analyzed manually to determine accuracy. Response times (RTs) were recorded automatically through SV-1 Voice Key (Cedrus). Incorrect trials were excluded in the subsequent analyses.

We applied logistic mixed-effects regression models to accuracy and linear mixed-effects models to RT using RStudio, including participants as a random effect (Bates et al., 2015). We investigated the effects of stimulation on RT for consistent words, inconsistent words and pseudowords by dividing the stimuli into three corresponding subsets. Additionally, we examined the interaction between stimulation and condition (consistent words, inconsistent words, pseudowords). To analyze the frequency effect, we divided the word data set (comprising consistent and inconsistent words) into two subsets based on a median split: low-frequency and high-frequency words. This allowed us to analyze the interaction effects between simulation, consistency, and frequency. Variables of no interest (number of letters, OLD20, frequency, concreteness, and valence) were added in the analysis as regressors.

For the Picture Plausibility task, log-transformed RT was also analyzed using a Bayesian linear mixed-effects model implemented in *brms* (Bürkner, 2017), with participants included as a random intercept. Priors were set as follows: fixed effects were centered at zero with *SD* = 0.04; the intercept was assigned a Student-t prior (*df* = 3, mean = 6.8, scale = 0.5), and both the random intercept and residual *SD* were assigned exponential priors (rate = 2). We estimated the model with four chains of 2,000 iterations each, with 1,000 warmup iterations.

We also performed paired *t*-test to evaluate the effect of stimulation on the Inverse Efficiency Score (IES), calculated as the mean RT divided by the mean Accuracy for each subject and stimulation type. This measure potentially better captures the speed-accuracy tradeoff, but has lower power than the mixed-effects model.

### Sample Size

The sample size for the Word Naming task was determined using G*power 3.1 for linear multiple regression. Using an effect size of 0.4 based on previous studies, with a statistical power of 0.80, alpha level of 0.05, the required sample size was calculated to be 32 participants.

## RESULTS

### Experiment 1 (ATL Word Naming Task)

There was no significant difference in RT and accuracy between real and sham conditions when merging all words and pseudowords (RT: *β* = 0.924, *SE* = 5.120, *t* = 0.180, *p* = 0.857; Accuracy: *β* = 0.108, *SE* = 0.167, *z* = 0.647, *p* = 0.518). Specifically, in the sham condition, the mean RT were 945.1 ms (*SD* = 290.6 ms) and in the real condition, they were 952.2 ms (*SD* = 299.1 ms) collapsing across words and pseudowords.

**Figure 3.**
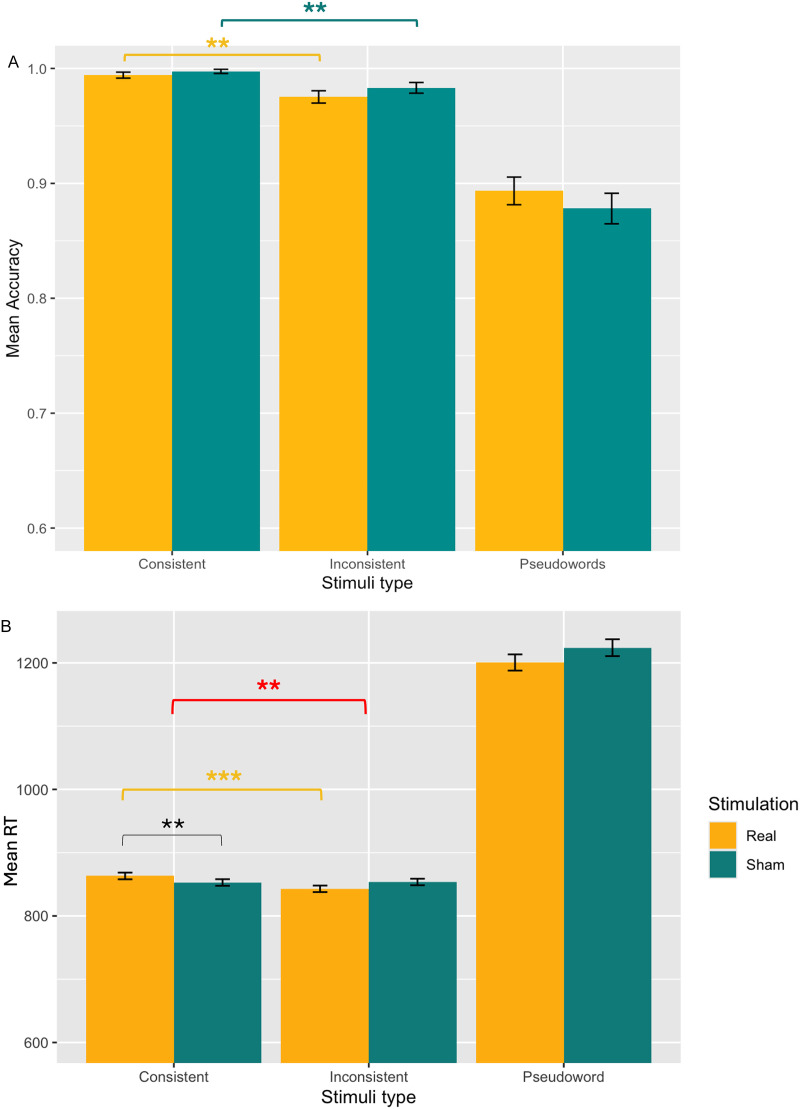
Mean accuracy (A) and RT (B) in the Word Naming task with real (yellow) and sham (blue) stimulation for consistent and inconsistent words. Error bars represent 95% confidence intervals. ***p < 0.001, **p < 0.01.

Regarding pseudowords, we found no effect of stimulation on RT (*β* = 12.416, *SE* = 14.853, *t* = 0.836, *p* = 0.403) or Accuracy (*β* = −0.060, *SE* = 0.199, *z* = 0.304, *p* = 0.761), regressing out other variables. We then investigated the difference between consistent and inconsistent words (Figure 3). In the sham condition, while there was no significant difference on RT between consistent and inconsistent words (*β* = 0.382, *SE* = 6.670, *t* = 0.057, *p* = 0.954), significant differences on accuracy revealed that inconsistent words were read aloud less accurately compared to consistent words (*β* = −2.593, *SE* = 0.843, *z* = −3.075, *p* = 0.002) (Figure 3). In the real condition, significant differences between consistent and inconsistent words were found for both RT (*β* = −26.448, *SE* = 6.653, *t* = −3.975, *p* < 0.001) and accuracy (*β* = −1.935, *SE* = 0.644, *z* = −3.006, *p* = 0.003) (Figure 3). Specifically, inconsistent words were named more quickly (Mean diff = 20.3 ms) but less accuracy (Mean diff = 0.02) compared to the consistent words. Importantly, there is a significant interaction effect of stimulation and consistency on RT (*β* = 24.856, *SE* = 8.813, *t* = 2.820, *p* = 0.0048), after regressing out all other variables (i.e., number of letters, frequency, OLD20, concreteness, valence). This indicated that the ATL stimulation facilitated the naming of the inconsistent words, whereas it induced an inhibitory effect on the naming of the consistent words (Figure 3).

**Figure 4.**
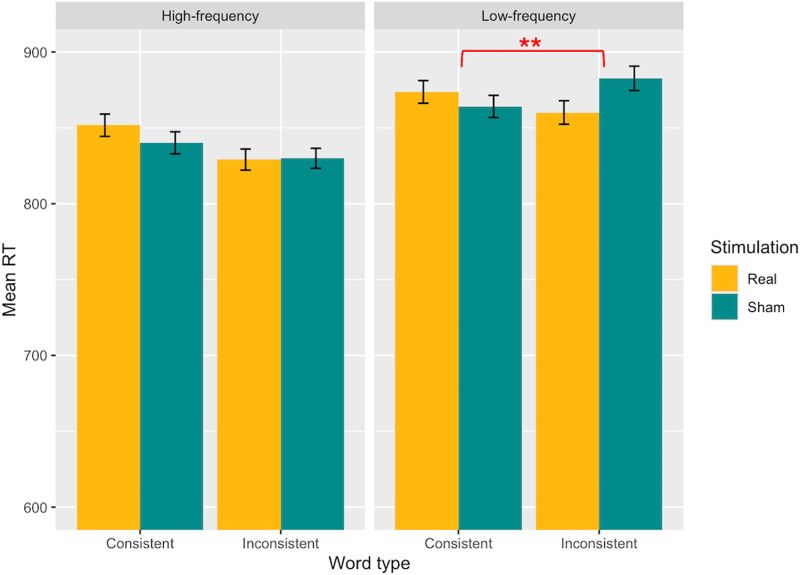
RT in the Word Naming task with real (yellow) and sham (blue) stimulation for consistent and inconsistent words, with the median-split dividing into high-frequency and low-frequency categories. Error bars represent 95% confidence intervals. **p < 0.01.

Using a median split, we then divided the words into high-frequency and low-frequency categories for both consistent and inconsistent words (Figure 4). In the high-frequency subset, there was no interaction effect of stimulation and consistency on Accuracy or RT (Accuracy: *β* = −0.976, *SE* = 99.622, *z* = −0.010, *p* = 0.992; RT: *β* = 15.290, *SE* = 11.823, *t* = 1.293, *p* = 0.196).^1^ In contrast, the low-frequency subset showed an interaction effect of consistency and stimulation on RT (*β* = 36.451, *SE* = 13.137, *t* = 2.775, *p* = 0.006), but no effect on Accuracy (*β* = −0.010, *SE* = 1.019, *z* = −0.010, *p* = 0.992) after regressing out all other variables. ATL stimulation resulted in this interaction by facilitating the naming of inconsistent low-frequency words, while inhibiting the naming of consistent low-frequency words.

We observed a significant interaction effect on RT between frequency and consistency in the sham subset (*β* = 25.403, *SE* = 12.510, *t* = 2.031, *p* = 0.042), after controlling for all other variables as regressors (i.e., number of letters, OLD20, concreteness, valence). However, in the real stimulation subset, there was no interaction between frequency and consistency on RT (*β* = 5.193, *SE* = 12.578, *t* = 0.413, *p* = 0.680). This finding held even when regressors of no interest were included in the analysis, indicating that ATL stimulation modulated the influence of frequency and consistency on naming Accuracy and RT. Specifically, ATL stimulation appears to reduce the influence of frequency-consistency interaction that was found in the sham condition.

**Figure 5.**
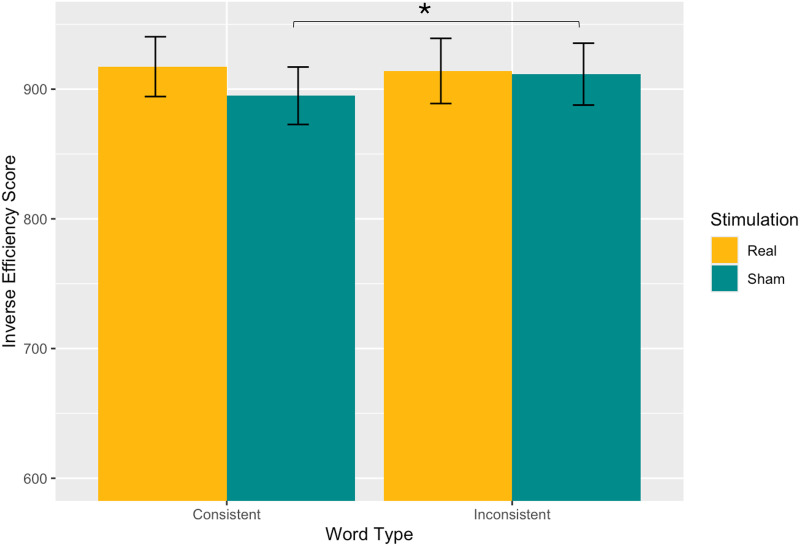
Inverse Efficiency Score (RT/accuracy) in the Word Naming task with real (yellow) and sham (blue) stimulation for consistent and inconsistent words. Error bars represent 95% confidence intervals. *p < 0.05.

For sham stimulation, we found a significant effect of consistency, with the IES being higher for inconsistent words compared to consistent words (*t* = −2.39, *p* = 0.026, mean diff = −16.662, CI = [−31.155; −2.169]) (Figure 5). For real stimulation, this difference was not significant (*t* = 0.211, *p* = 0.834, mean diff = 3.311, CI = [−29.088; 35.710]). This indicates that the consistency effect, which led to better performance for consistent words, disappeared with ATL stimulation.

### Experiment 2 (ATL Picture Plausibility Task)

**Figure 6.**
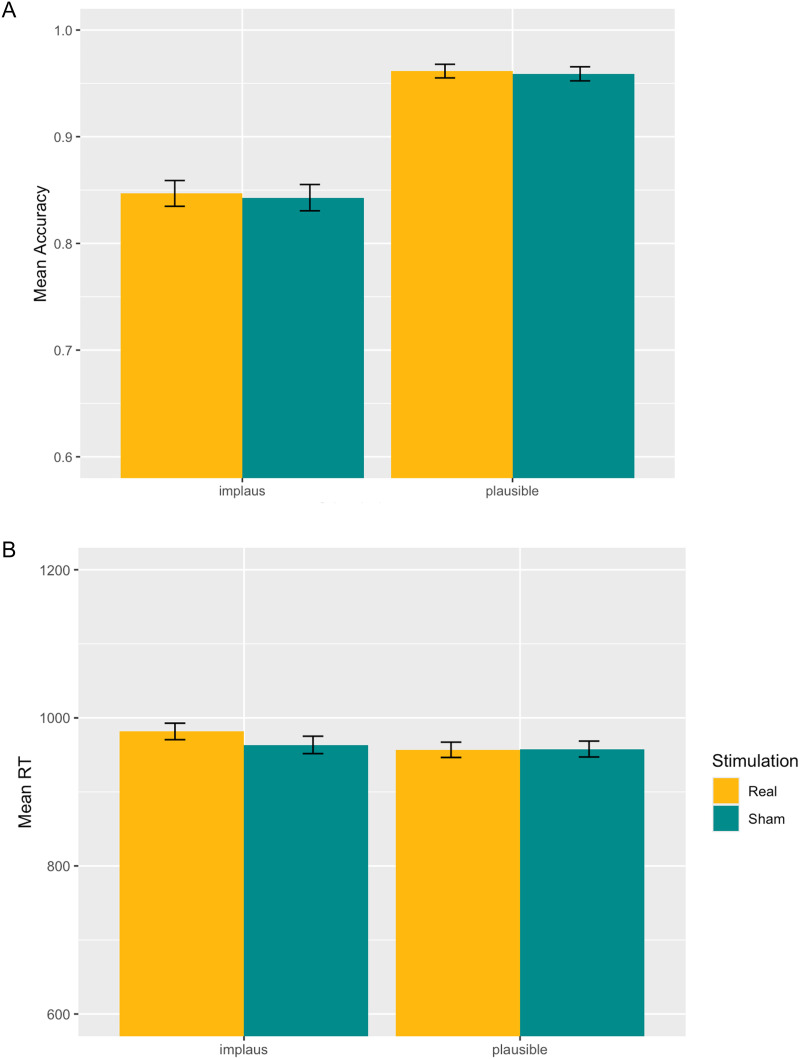
Mean accuracy (A) and RT (B) in the Picture Plausibility task with real (yellow) and sham (blue) stimulation targeting the ATL. Error bars represent 95% confidence intervals.

In the Picture Plausibility task, when stimulation targeting the ATL (Figure 6), we found no significant effect of stimulation on both RT and accuracy, when controlling for condition effect (implausible vs. plausible) (RT: *β* = −5.397, *SE* = 9.845, *t* = −0.548, *p* = 0.584; Accuracy: *β* = −0.038, *SE* = 0.116, *z* = −0.328, *p* = 0.743). When subdividing the data between plausible and implausible stimuli, we found no effect on the plausible subset (RT: *β* = 5.412, *SE* = 13.281, *t* = 0.408, *p* = 0.684; Accuracy: *β* = −0.067, *SE* = 0.240, *z* = −0.28, *p* = 0.779) and the implausible subset (RT: *β* = −19.18, *SE* = 14.52, *t* = −1.321, *p* = 0.187; Accuracy: *β* = −0.031, *SE* = 0.132, *z* = −0.241, *p* = 0.81). This suggests that ATL stimulation did not modulate the non-lexical semantic processing.

Bayesian statistics were consistent with these results, showing no evidence for an effect of stimulation on logRT when controlling for condition (implausible vs. plausible), as credible interval (Crl) includes zero (*β* = −0.01, 95% CrI [−0.03; 0.01]). Similarly, after subdividing the data by stimulus type, we found no evidence for an effect of stimulation on logRT (plausible subset: *β* = 0.00, 95% CrI [−0.02, 0.03]; implausible subset: *β* = −0.02, 95% CrI [−0.05, 0.00]).

### Experiment 3 (TOC Picture Plausibility task)

**Figure 7.**
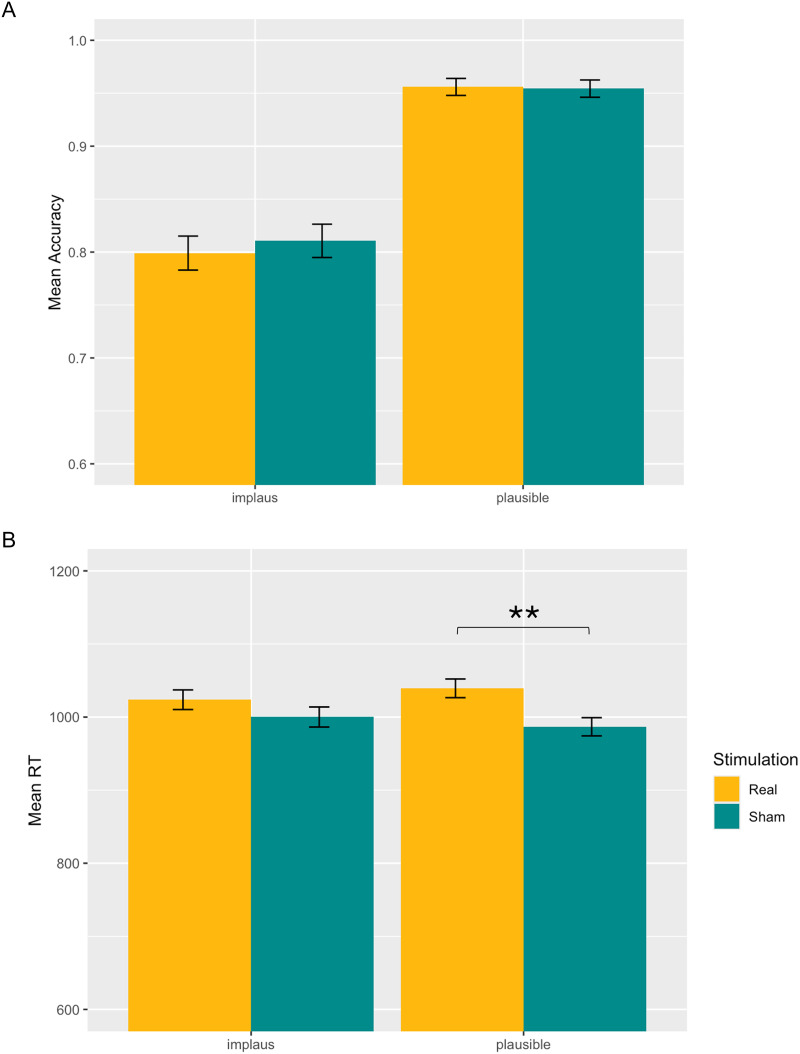
Mean accuracy (A) and RT (B) in the Picture Plausibility task with real (yellow) and sham (blue) stimulation targeting the TOC. Error bars represent 95% confidence intervals. **p < 0.01.

When stimulating the TOC, a significant effect of stimulation was observed on RT (*β* = −39.19, *SE* = 11.97, *t* = −3.274, *p* = 0.001) when controlling for stimulus condition in the Picture Plausibility task, with real stimulation inducing longer RT compared to the sham condition (Figure 7). There were no significant differences in accuracy (*β* = 0.045, *SE* = 0.126, *z* = 0.359, *p* = 0.72). In the plausible subset, we found a significant effect on RT (*β* = −49.89, *SE* = 16.38, *t* = −3.043, *p* = 0.002), but no effect on Accuracy (*β* = −0.040, *SE* = 0.268, *z* = −0.15, *p* = 0.881) and no effect on the implausible pictures (RT: *β* = −24.51, *SE* = 17.32, *t* = −1.415, *p* = 0.157; Accuracy: *β* = 0.068, *SE* = 0.144, *z* = 0.473, *p* = 0.636). This result validated the Picture Plausibility task, demonstrating that the non-lexical semantic processing was indeed modulated by the TOC.

Using Bayesian statistics, we found consistent results, indicating evidence for an effect of stimulation on logRT when controlling for condition (implausible vs. plausible) (*β* = −0.04, 95% CrI [−0.06; −0.02]). Similarly, after subdividing the data by stimulus type, we found evidence for an effect of stimulation on logRT, that was stronger for plausible items (plausible subset: *β* = −0.05, 95% CrI [−0.07, −0.02]). For implausible subset, the estimated effect was also negative, but the 95% credible interval included zero, indicating greater uncertainty (implausible subset: *β* = −0.03, 95% CrI [−0.06, 0.00]).

### Carry-Over Effect Control

We applied logistic and linear mixed effect model to control any potential order effect by evaluating the interaction between Stimulation Order (Sham/Real vs. Real/Sham) and Stimulation Type (Sham vs. Real) in the subset of participants who had a 24-hr gap between sessions.

In the Experiment 1 (*n* = 5), we did not find an interaction effect on Accuracy or RT when all stimuli were merged (Accuracy: *β* = −2.152, *SE* = 2.933, *z* = −0.734, *p* = 0.463; RT: *β* = −25.923, *SE* = 103.087, *t* = −0.251, *p* = 0.818), nor when divided by stimuli type: consistent (Accuracy: *β* = 21.341, *SE* = 760.672, *z* = 0.028, *p* = 0.978; RT: *β* = 19.431, *SE* = 144.680, *t* = 0.134, *p* = 0.902); inconsistent (Accuracy: *β* = −17.765, *SE* = 128.505, *z* = −0.138, *p* = 0.890; RT: *β* = 31.646, *SE* = 146.992, *t* = 0.215, *p* = 0.843); pseudowords (Accuracy: *β* = −2.148, *SE* = 4.206, *z* = −0.511, *p* = 0.610; RT: *β* = −211.759, *SE* = 224.595, *t* = −0.943, *p* = 0.416). Similarly, no interaction effect on RT or Accuracy was found for Experiment 2 (*n* = 11) (Accuracy: *β* = 0.648, *SE* = 0.450, *z* = 1.440, *p* = 0.150; RT: *β* = 150.648, *SE* = 176.974, *t* = 0.851, *p* = 0.417) or Experiment 3 (*n* = 7) (Accuracy: *β* = 0.387, *SE* = 0.645, *z* = 0.600, *p* = 0.549; RT: *β* = −17.610, *SE* = 297.598, *t* = −0.059, *p* = 0.955). These results highlight that the present findings were not driven by an order effect.

## DISCUSSION

We examined the role of ATL in processing spelling-sound consistency, and the role of semantics, in three experiments. Surface alexia is associated with ATL atrophy and semantic deficits. At the same time, several studies suggest a dissociation between surface alexia and semantic deficits. Our results have direct implications for this debate.

First, in the sham condition, we replicated the classic consistency × frequency interaction, whereby low-frequency inconsistent words were read aloud slower than high-frequency inconsistent words (Plaut et al., 1996; Seidenberg et al., 1984). Frequency did not have an impact on reading consistent words. Applying stimulation to the left ATL eliminated this interaction. Low-frequency words showed a stimulation × consistency interaction, while the high-frequency words did not. The disadvantage of low-frequency inconsistent words was eliminated by ATL stimulation. These results support the causal role of ATL in reading, and especially for reading inconsistent words.

ATL stimulation induced changes in RT for words but not for pseudowords, supporting the idea that reading words and pseudowords involves at least partially distinct brain processes. Models of reading suggest a phonological/sublexical pathway for reading pseudowords, sometimes termed the ‘dorsal pathway’ (Graves et al., 2010; Jobard et al., 2003; Mechelli et al., 2003; Taylor et al., 2013). This pathway is thought to include supramarginal gyrus and posterior temporal gyri. These results suggest that ATL is not part of this pathway, but rather part of a ventral lexicosemantic pathway (Hoffman et al., 2015; Provost et al., 2016; Wilson et al., 2012).

The central question then relates to what specific role ATL plays in reading. ATL may support lexical and/or semantic processes. If it has a semantic role, it would be consistent with findings of semantic variant of PPA patients who also have surface alexia. To test this hypothesis, Experiment 2 employed a nonverbal semantic task. This task clearly requires accessing concepts and making semantic judgments but does not have explicit verbal demands. A commonly used task in aphasia to examine nonverbal semantics is the picture versions of Pyramids and Palm Trees Test, and Kissing and Dancing Test. Both tasks involve significant executive demands in addition to semantic judgment, as they require finding and comparing relationships between three stimuli. To reduce these executive demands, the Picture Plausibility task was designed to have a single stimulus item on each trial. While all tasks involve some degree of executive demands, processing a single image rather than three likely places lower demands on executive processing, as comparisons between pairs of images is eliminated. Experiment 2 indicated that ATL stimulation had no impact on the performance of this task. This result provides evidence against the idea that the primary role of ATL in reading is related to the storage of semantic representations.

While Experiment 2 had sufficient power to detect small effects, it nonetheless provided a negative result. To test that the Picture Plausibility task is indeed sensitive to semantics, Experiment 3 stimulated TOC, an area known to support higher order visual semantics. Here, we found clear effects of stimulation on the Picture Plausibility task. One dimension that ATL is sensitive to is category typicality. For example, Rogers et al. (2015) highlighted the role of the ATL in processing atypical items in individuals with svPPA and bilateral ATL atrophy. While we did not systematically manipulate typicality dimensions, most plausible items in the PP task were typical. However, a semantic hub is expected to be sensitive to typical objects as well, even if it shows greater activation for atypical items in some tasks; this was not found here. Another relevant study is an Hauk et al. (2007), who used EEG with inauthentic and authentic items that are very similar to the implausible and plausible items in the Picture Plausibility task (e.g., one inauthentic item depicts a fox with a hump like a camel, similar to the implausible objects in the present study, where objects and parts are mismatched). These inauthentic objects had greater source activation across most of the temporal lobe, including the TOC and ATL. In our results, when the stimuli were divided by plausibility (i.e., implausible vs. plausible items), only the plausible subset showed significant effects following TOC stimulation, and neither showed any effect of ATL stimulation. This pattern may be explained by the fact that plausible items elicit richer or more coherent semantic activation, whereas implausible items are harder to interpret and generate less semantic activation. Overall, these results are consistent with a large body of previous findings suggesting that TOC is sensitive to visual semantics, as it can detect the categorical structure of objects and/or the activation of semantic features (Bracci et al., 2015; Fairhall & Caramazza, 2013; Hauk et al., 2007; Kable et al., 2002; Kellenbach et al., 2003; van Dam et al., 2019).

An rTMS study by Ueno et al. (2018) targeting the left ventral ATL is noteworthy here. It induced regularization errors in reading low-frequency inconsistent words in neurotypical Japanese adults. The difference in the direction of effects between our and Ueno et al. (2018) study can be explained by the stimulation methodology (Quartarone et al., 2006). They applied low-frequency inhibitory rTMS, whereas we used high-density anodal tDCS, which is generally expected to induce facilitatory effects. Ueno et al. (2018) interpreted their findings as evidence of the ATL’s role in semantic processing during the reading of low-frequency inconsistent words. However, this claim was not tested using a nonverbal semantic task.

A meta-analysis focusing on the role of the ATL in semantic processing in functional neuroimaging studies argues that the ATL functions as an amodal semantic system (Visser et al., 2010), as this region was activated even during non-verbal stimuli (pictures) in neurotypical participants. However, the meta-analysis examined the effect of pictures used as *stimuli*, which does not rule out the presence of a verbal component in the tasks, such as picture naming, the most common paradigm used with pictures. Similarly, the meta-analysis of Rice et al. (2015b) compared the role of both ATLs in processing conceptual knowledge across non-verbal and verbal stimulus modalities, showing ATL activation even for the non-verbal modality. However, non-verbal tasks were defined based on input modality, including again several studies with picture naming tasks. Moreover, some studies in both meta-analyses (Rice, Lambon Ralph, et al., 2015; Visser et al., 2010) involved tasks with unique entities (e.g., famous people), which are known to implicate the ATL. Aligning with this, a study combining three fMRI datasets (Rice et al., 2018) comparing activation across concepts probed with pictures or written words, showed a ventral and lateral ATL activation even for non-verbal stimuli. However, both verbal and non-verbal tasks involved unique entities, potentially explaining ATL activation. In the meta-analysis by Visser et al. (2010), activation for picture-based tasks relative to verbal tasks was found in the medial half of the ATL, whereas the lateral ATL was targeted here. Finaly, temporal lobe epilepsy people with right or left anterior temporal lobectomy exhibited mild semantic impairments in non-verbal tasks, with graded differences such that left lobectomy was associated with greater impairment in naming and written word-based tasks (Rice et al., 2018; Rouse et al., 2024). However, temporal lobe epilepsy involves a potential functional reorganization, which might explain the relative mild impairments. In addition, these resections primarily focused on the medial part of the ATL, whereas the present study stimulated the lateral ATL. Electrocorticography studies also tend to show semantic effects in more medial and posterior sections of the ATL compared to the region stimulated in the present study (Shimotake et al., 2015). Hence, the current results are potentially compatible with this body of literature. Furthermore, two studies (Pobric et al., 2010; Vandenberghe et al., 1996) showed that a nonverbal semantic task was affected by ATL stimulation or activated ATL more. These results are potentially contradictory to our findings. However, both studies used the Pyramids and Palm Trees, Kissing and Dancing, Camels and Cactus Tests, or similar tasks, which in addition to semantic processing, may place greater demands on reasoning and executive processing by requiring comparison and selection of the most plausible associate among two or three alternatives. For example, a *shotgun* is not a typical associate of the target *rabbit*, but given a choice of *shotgun* and *handgun*, one might reason that a shotgun could be related to rabbit in the context of hunting, whereas handguns are not typically used for hunting or in other scenarios related to rabbits. Such reasoning may also be verbally mediated, which is not the case for the control tasks in these studies (i.e., the scrambled picture and physical size-matching tasks). On the other hand, it should be noted that although these types of tasks may be verbally mediated, they do not appear to place strong demands on core language processes such as phonology or speech fluency (Halai et al., 2017), mitigating this concern. We developed the Picture Plausibility task to minimize such executive and reasoning demands, although this novel task requires further replication. Here we hypothesize that activations found in ATL in few studies that used such tests are due to extra demands placed by these tasks, and this suggestion also remains to be tested in future studies. Taking into consideration these limitations in studies for the involvement of ATL in non-verbal semantic processing, Visser et al. (2011) showed activation of the ATL in a non-verbal semantic task, involving processing single stimuli that were not unique entities, and did not involve a lexical component either in the input or the output. The activation was mainly seen in the medial aspect of the ATL, with a small cluster on the ventrolateral surface, which could be considered as diverging from the present findings and requires further investigation.

Atrophy in the temporal lobe, including ATL, is associated with both verbal and nonverbal semantic tasks in PPA (Adlam et al., 2006; Butler et al., 2009; Jefferies & Lambon Ralph, 2006; Patterson et al., 2007). As instance, Butler et al. (2009) found that bilateral ATL atrophy was corelated with semantic impairments across modalities. How can this be reconciled with the present findings? In PPA, atrophy in patients who exhibit semantic deficits is not restricted to ATL, but tends to be widespread, including middle and posterior parts of the lateral and ventral temporal lobes, while the present study only stimulated the lateral ATL. Authors often emphasize the site of peak atrophy, but the full extent of atrophy is much more widespread than the peak. Hurley et al. (2012) compared two groups of PPA patients distinguished by word comprehension impairments. The PPA-S group (i.e. semantic variant of PPA) was severely impaired in word comprehension, while PPA-GL (i.e., agrammatic and logopenic variant) was not. Both groups performed well in a nonverbal picture categorization task. PPA-S patients had greater atrophy than PPA-GL patients in the left ATL and were impaired in all lexical tasks (picture naming, word-to-picture pointing, and word categorization) but could perform a nonverbal semantic task (picture categorization). Similarly, Mesulam et al. (2013) reported that in a cohort of PPA participants with relatively restricted atrophy in the left ATL, impairments were seen in object naming and word associations tasks, but not in picture-picture association tasks. Such findings are consistent with the present results, where the left ATL is important for lexical, but not nonverbal semantic, processing.

What is a possible role of ATL that is relevant to lexical tasks, if that role is not primarily semantic? We suggest that ATL is a site of units that map between orthographic/phonological codes and semantic representations but does not store semantic representations itself. These units are more likely to be impaired (with atrophy or with noninvasive stimulation) when the mapping is inconsistent, and words are low-frequency. In Dell’s interactive two-step model (Dell et al., 1997, 1999), two layers represent semantic feature and phonological codes. An intermediate layer, named “words,” contains precisely this type of units that provide a mapping between semantics and phonology, but do not contain integrated semantics or phonology themselves. We propose that ATL is a candidate site for such units. This account explains results where the relationship between alexia and semantic impairments is inconsistent (Playfoot et al., 2018). Surface alexia can be observed without semantic impairments (Binder et al., 2016; Gvion & Friedmann, 2016), and semantic impairments do not necessarily lead to surface alexia (Blazely et al., 2005; Cipolotti & Warrington, 1995). Indeed, surface alexia results from ATL damage that leads to lexical, but not semantic, impairment. However, it is worth noting that in the lesion-symptom mapping study by Binder et al. (2016), most of the ventral ATL was poorly covered by lesion overlap. The regularization errors observed could result from ATL disconnection rather than direct ATL damage (Binder et al., 2016). Furthermore, some studies hypothesize that inconsistent word reading errors might be linked to phonological deficits, as inconsistent words activate several phonological competitors (Woollams & Patterson, 2012). These phonological deficits can induce similar errors in the reading of pseudowords. However, our results suggest a clear dissociation between word and pseudoword reading, as HD-tDCS modulated only word reading.

We note that our results do not necessarily show that semantic information is not needed or not accessed for reading inconsistent words. However, our results do suggest that if such information is needed, left lateral ATL is not the site of its storage. It is entirely possible that other areas, including TOC, may contain semantic representations that are accessed to a greater extent for reading inconsistent words. Experiment 3 in our study was meant only to test the sensitivity of the Picture Plausibility task and did not contain the reading task. Future experiments with reading, other lexical tasks, and non-lexical semantic tasks that stimulate TOC and other sites associated with semantics may shed light on this question.

Another potential explanation for the lack of left ATL stimulation effect in the Picture Plausibility task, a nonverbal and explicitly semantic task, is the possibility of graded modality specialization between the two ATLs. Specifically, each ATL may be differentially sensitive to a specific stimulus modality, with one hemisphere showing greater responsiveness to verbal stimuli and the other to nonverbal or visual inputs (e.g., Lambon Ralph et al., 2001; Mion et al., 2010; Rice, Hoffman, et al., 2015). Hence, a promising future direction is to use these tasks with right ATL and/or bimodal stimulation targeting both ATLs to test the roles of both hemispheres with respect to modality (Lambon Ralph et al., 2017; Rice, Lambon Ralph, et al., 2015).

Finally, the directionally of effects in Experiment 1 vs. Experiment 3 were different. As opposed to traditional tDCS, the interpretation of ‘anodal’ and ‘cathodal’ stimulation in HD-tDCS is not straightforward (Garnett et al., 2015). Facilitatory or inhibitory effects may be obtained in HD-tDCS based on a number of factors, as results may be influenced by inter-individual variability, orientation of neuron populations relative to the current flow, and the fact that parameters of stimulation are based on effects seen in studies of the motor cortex (Li et al., 2015; Quartarone et al., 2006; Rahman et al., 2013). One hypothesis for this finding is that tasks with higher cognitive demands may be more susceptible to stimulation, which could explain the main effect observed between low-frequency words, as highlighted by previous work on working memory (Berryhill et al., 2014; Framorando et al., 2021). Consistent with this view, Wang et al. (2021) reported opposite effects (facilitation vs. inhibition) of anodal tDCS targeting the dorsolateral prefrontal cortex depending on task difficulty (i.e., facilitation in the parity/magnitude task but inhibition in the parity/vowel-consonant task). Another explanation for this differential effect is that identical stimulation can produce either inhibitory or facilitatory effects depending on the orientation of the neuronal populations relative to the current flow (Li et al., 2015; Rahman et al., 2013). Since the Word Naming and Picture Plausibility tasks engage different cognitive processes, the same stimulation may have induced differential effects across tasks.

One limitation of the present study concerns stimulation of the TOC. Given that HD-tDCS may also have affected the visual cortex, the observed effects on the Picture Plausibility task could partly reflect modulation of lower-level visual processing, even though the strongest focus of stimulation was on the occipito-temporal cortex and not on the primary visual cortex. Including a visual non-semantic control task would have helped control for this potential confound.

## CONCLUSIONS

HD-tDCS stimulation of the left lateral ATL had a differential effect on consistent and inconsistent words. The frequency × consistency interaction, where low-frequency inconsistent words are the slowest, was eliminated by this stimulation. Simulation × consistency interaction was seen for low-frequency words. This supports a causal role of left lateral ATL in reading inconsistent words, similar to what is seen in PPA patients with surface alexia. The same ATL stimulation had no effect on a nonverbal semantic task, suggesting that the role of the left lateral ATL in reading may be related to lexical rather than to amodal semantic processing. This can explain several reports where surface alexia is dissociated from sematic impairments. The results are also consistent with a view suggesting graded specialization between the left and right ATLs for verbal and visual stimuli, respectively. In both cases, the present findings elucidate the role of the left ATL in reading.

## FUNDING INFORMATION

Rutvik H. Desai, NIH/NIDCD, Award ID: R01DC017162.

## Author contributions

Experiment design RHD, KJ, NR; data collection SAP, CX, SC, KJ; data analysis SAP, CX, SC; manuscript writing SAP, CX, RHD, KJ, NR.

## DATA AND CODE AVAILABILITY STATEMENT

Data and code for this study are available on OSF: https://osf.io/2nmcr/overview?view_only=cb893f6a012e410a8eae83b714001267.

## Notes

^1^ The accuracy model failed to converge. Therefore, a simplified model was fitted, including interaction terms (Stimulation × Consistency) and participants as a random effect, but excluding additional regressors.
